# 2-{2,4,6-Tris(bromo­meth­yl)-3,5-bis­[(1,3-dioxoisoindolin-2-yl)meth­yl]benz­yl}iso­indoline-1,3-dione toluene monosolvate

**DOI:** 10.1107/S1600536814004383

**Published:** 2014-03-05

**Authors:** Niklas Koch, Wilhelm Seichter, Monika Mazik

**Affiliations:** a Institut für Organische Chemie, echnische Universität Bergakademie Freiberg, Leipziger Strasse 29, D-09596 Freiberg/Sachsen, Germany

## Abstract

In the title compound, C_36_H_24_Br_3_N_3_O_6_·C_7_H_8_, the toluene solvent mol­ecule is associated with the receptor mol­ecule *via* C—H⋯π bonding. The planes of the phthalimido groups are inclined at 77.0 (1), 63.0 (1) and 77.8 (1)° with respect to the benzene ring. The mol­ecular conformation is stabilized by C—H⋯O and C—H⋯Br hydrogen bonds. The crystal structure features non-classical hydrogen bonds of the C—H⋯N, C—H⋯O and C—H⋯Br type, leading to a three-dimensional cross-linking of molecules. The pattern of non-covalent inter­molecular bonding is completed by O⋯Br halogen bonds [3.306 (3) Å], which link the receptor mol­ecules into infinite strands extending along the *a*-axis direction.

## Related literature   

For heteroditopic receptors and their applications, see: McConnell & Beer (2012 ▶); Kirkovits *et al.* (2001 ▶); Kinnear *et al.* (1994 ▶); Hossain & Schneider (1998 ▶); Tsukube *et al.* (1999 ▶); Smith (2010 ▶). For C—H⋯π inter­actions, see: Nishio *et al.* (2009 ▶). For non-classic hydrogen bonds, see: Desiraju & Steiner (1999 ▶). For halogen bonding, see: Metrangolo & Resnati (2008 ▶). For the synthesis and use of the title compound, see: Roelens *et al.* (2009 ▶).
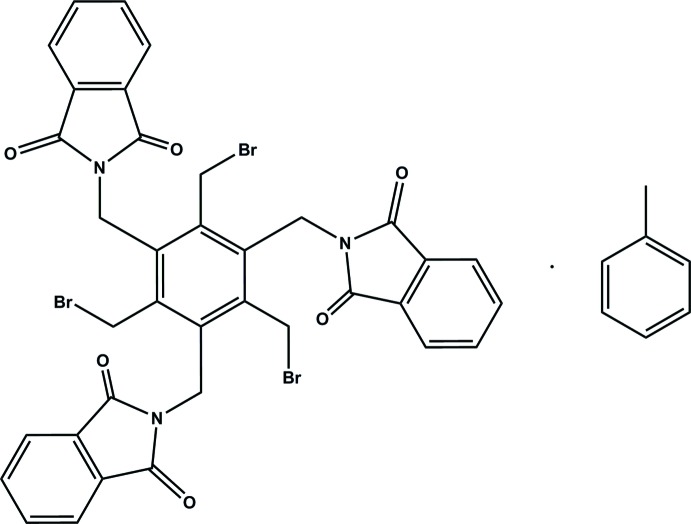


## Experimental   

### 

#### Crystal data  

C_36_H_24_Br_3_N_3_O_6_·C_7_H_8_
*M* *_r_* = 926.45
Orthorhombic, 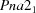

*a* = 9.2879 (2) Å
*b* = 39.2730 (11) Å
*c* = 10.5592 (3) Å
*V* = 3851.61 (17) Å^3^
*Z* = 4
Mo *K*α radiation
μ = 3.20 mm^−1^
*T* = 100 K
0.50 × 0.42 × 0.34 mm

#### Data collection  

Bruker APEXII CCD area-detector diffractometer
Absorption correction: multi-scan (*SADABS*; Bruker, 2008 ▶) *T*_min_ = 0.298, *T*_max_ = 0.410
59694 measured reflections
10997 independent reflections
10267 reflections with *I* > 2σ(*I*)
*R*_int_ = 0.029

#### Refinement  

*R*[*F* ^2^ > 2σ(*F* ^2^)] = 0.026
*wR*(*F* ^2^) = 0.057
*S* = 1.06
10997 reflections
482 parameters
1 restraint
H-atom parameters constrained
Δρ_max_ = 0.41 e Å^−3^
Δρ_min_ = −0.43 e Å^−3^
Absolute structure: Flack (1983 ▶), 5205 Friedel pairs
Absolute structure parameter: 0.015 (4)

### 

Data collection: *APEX2* (Bruker, 2008 ▶); cell refinement: *SAINT* (Bruker, 2008 ▶); data reduction: *SAINT*; program(s) used to solve structure: *SHELXS97* (Sheldrick, 2008 ▶); program(s) used to refine structure: *SHELXL97* (Sheldrick, 2008 ▶); molecular graphics: *ORTEP-3 for Windows* (Farrugia, 2012 ▶); software used to prepare material for publication: *SHELXL97*.

## Supplementary Material

Crystal structure: contains datablock(s) I, New_Global_Publ_Block. DOI: 10.1107/S1600536814004383/xu5768sup1.cif

Structure factors: contains datablock(s) I. DOI: 10.1107/S1600536814004383/xu5768Isup2.hkl

Supporting information file. DOI: 10.1107/S1600536814004383/xu5768Isup3.cml

CCDC reference: 988768

Additional supporting information:  crystallographic information; 3D view; checkCIF report

## Figures and Tables

**Table 1:** Hydrogen-bond geometry (Å, °) *Cg*1 and *Cg*2 are centroids of the C1*A*–C6*A* and C21–C26 benzene rings, respectively.

*D*—H⋯*A*	*D*—H	H⋯*A*	*D*⋯*A*	*D*—H⋯*A*
C7—H7*B*⋯O3	0.99	2.47	3.309 (3)	142
C8—H8*A*⋯O4	0.99	2.18	3.061 (3)	147
C8—H8*B*⋯O5	0.99	2.36	3.036 (3)	125
C8—H8*B*⋯O6^i^	0.99	2.58	3.477 (3)	151
C9—H9*A*⋯O1	0.99	2.54	3.308 (3)	135
C10—H10*B*⋯Br3	0.99	2.77	3.519 (2)	133
C14—H14⋯O5^ii^	0.95	2.49	3.080 (3)	121
C19—H19*A*⋯Br1	0.99	2.89	3.642 (2)	133
C24—H24⋯O2^iii^	0.95	2.43	3.290 (3)	150
C28—H28*A*⋯O6^i^	0.99	2.58	3.344 (2)	134
C33—H33⋯Br2^iv^	0.95	2.77	3.590 (3)	145
C22—H22⋯*Cg*1^v^	0.95	2.66	3.599 (3)	169
C6*A*—H6*A*⋯*Cg*2	0.95	2.99	3.795 (3)	143

Symmetry codes: (i) 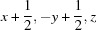; (ii) 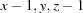; (iii) 
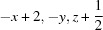
; (iv) 
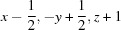
; (v) 
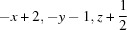
.

**Fig. 1.**
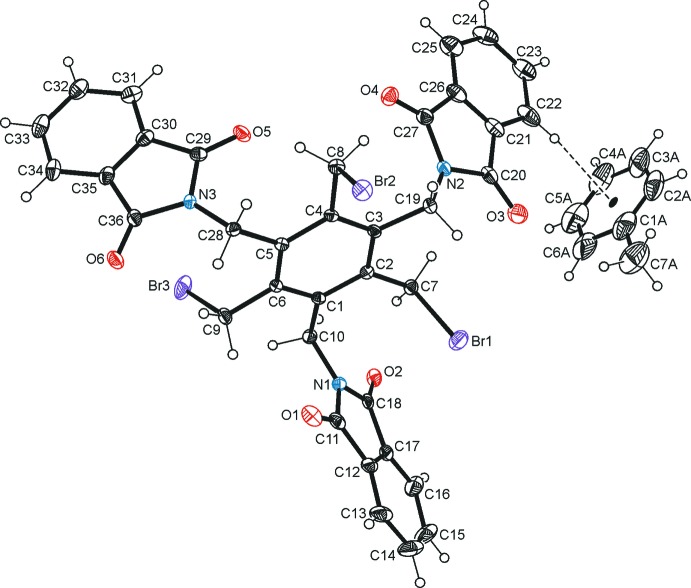
Perspective view of the molecular structure. Displacement ellipsoids of the non-H atoms are drawn at the 50% probability level.

**Fig. 2.**
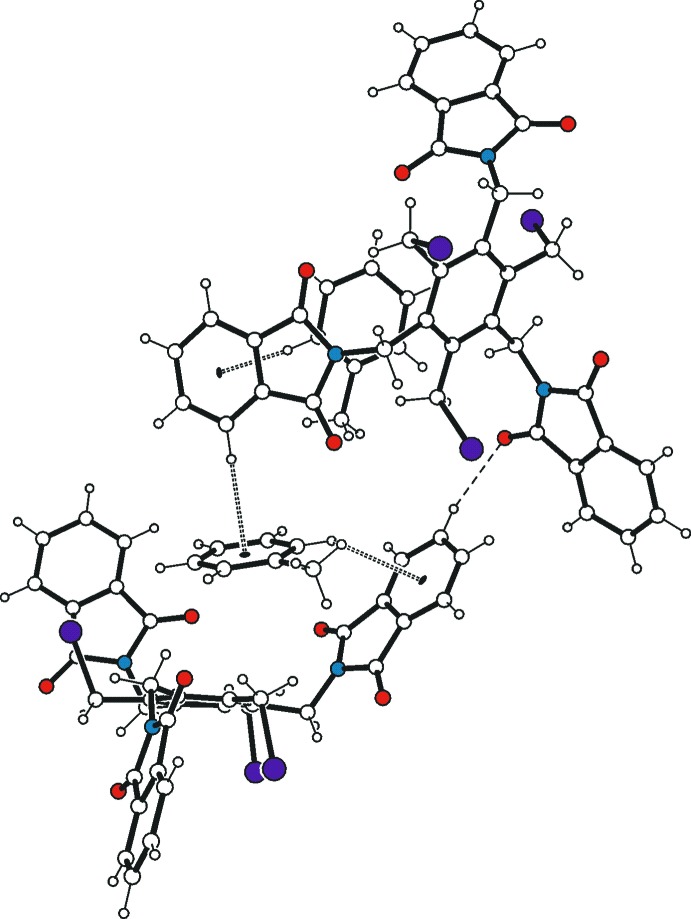
Packing motif of the crystal structure of the complex in which the C—H···O hydrogen bond is displayed as a broken line and C—H···π contacts as broken double lines.

**Fig. 3.**
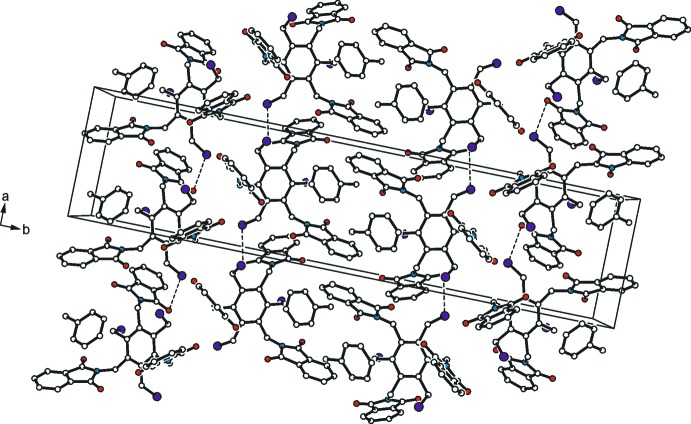
Packing diagram of the title compound viewed down the c axis. The hydrogen atoms are omitted for clarity. N atoms are displayed as blue, O atoms as red and bromine atoms as violet circles. The intermolecular contacts are marked by broken lines.

## supplementary crystallographic information

### 1. Comment

Our interest in the title compound, C_36_H_24_N_3_O_6_Br_3_, arises from its use as a precursor in the synthesis of novel heteroditopic artificial receptors containing a hexasubstituted benzene scaffold. The synthesis of ditopic receptors, which interact simultaneously with cationic and anionic guests in so called ion-pair recognition processes, is a rapidly developing area of research (McConnell & Beer, 2012). A number of ditopic receptors based on a macrocylic or acyclic scaffold have been designed and studied (Kinnear *et al.* (1994), Kirkovits *et al.* (2001), Hossain & Schneider (1998), Tsukube *et al.* (1999) and Smith (2010)) Interesting applications, such as binding of amino acids in their zwitterionic state, salt extraction and membrane transport, have been reported in the literature (for a recent review, see McConnell & Beer, 2012). In this context, the title compound is a particularly useful building block for the construction of a large number of receptors with different recognition units, because it provides a base for many synthetic modifications of the molecule structure. Crystallization of the title compound from toluene yields a 1:1 solvent complex (Fig. 1). According to the three-dimensional arrangement of the substituent groups around the periphery of the central arene ring, the conformational isomer of the receptor can be described as 1-down, 3,5-up tris((phthalimidomethyl), 2,4-down, 6-up tris(bromomethyl)benzene. The mean planes of the phthalimido moieties are inclined at angles of 77.0 (1), 63.0 (1) and 77.8 (1)° with reference to the plane of the benzene ring. The torsion angles given by the atomic sequences C1—C2—C7—Br1, C3—C4—C8—Br2 and C5—C6—C9—Br3 are 93.3 (2), 92.5 (2) and -99.8 (2) °, respectively. The host lattice is stabilized by non-conventional hydrogen bonds of the C—H···N [*d*(H···N) 2.55 Å], C—H···O [*d*(H···O) 2.18–2.58 Å] and C—H···Br type [*d*(H···Br) 2.77, 2.89 Å]. Moreover, the intermolecular distance O1···Br2 [3.306 (3) Å], which is less than the sum of van der Waals radii of the respective atoms [3.37 Å], indicates the presence of a weak C=O···Br halogen bond. As depicted in Fig. 2, the interactions between the receptor and the solvent molecule are reduced to weak C—H···π contacts with the aromatic ring of the toluene molecule and one of the phthalimido units of the receptor acting as acceptors [C22—H22···cg_1_ 2.66 Å, 169 °, C6A—H6A···cg_2_ 2.99 Å, 143 °]. Taking into account this kind of interactions, the crystal structure can be regarded as being composed of infinite strands of 1:1 complex units running along the crystallographic *a*-axis (Fig. 3).

### 2. Experimental

The synthesis of the title compound was carried out in a slightly modified literature procedure (Roelens *et al.* 2009). 1,3,5-Tris(phthalimidomethyl)-2,4,6-trimethylbenzene (1.00 g, 1.67 mmol) was dissolved in 1,2-dibromoethane (20 ml) and bromine (0.28 ml, 5.50 mmol) was added. The reaction mixture was stirred for overall 48 h under reflux and irradiation with a halogen bulb (500 W); after 24 h an additional amount of bromine (0.28 ml, 5.50 mmol) was added. The reaction mixture was cooled and washed with saturated aqueous solutions of sodium metabisulfite (2 *x* 20 ml), sodium bicarbonate (20 ml), and distilled water (20 ml). The organic phase was dried over sodium sulfate and the solvent removed by distillation. The residue was purified by column chromatography (SiO_2_, CH_2_Cl_2_, *R*_f_= 0.32) and the product obtained as an off-white solid. *Analysis data*: m.p. > 467 K; ^1^H NMR (400 MHz, CDCl_3_) δ 7.87–7.77 (m, 6H, aryl), 7.75–7.67 (m, 6H, aryl), 5.10 (s, 12H CH_2_); ^13^C NMR (100 MHz, CDCl_3_) δ 29.26, 36.40, 123.48, 132.03, 134.18, 134.87, 140.32, 168.28; EI—MS (70 eV) *m/z*: 595, 675, 754.

### 3. Refinement

H atoms were positioned geometrically and allowed to ride on their respective parent atoms, with C—H = 0.95 Å and *U*_iso_(H) = 1.2 *U*_eq_(C) for aryl, C—H = 0.99 Å and *U*_iso_(H) = 1.2 *U*_eq_(C) for methylene and C—H = 0.98 Å and *U*_iso_(H) = 1.5 *U*_eq_(C) for methyl.

### Crystal data

**Table:**

C_36_H_24_Br_3_N_3_O_6_·C_7_H_8_	*F*(000) = 1856
*M**_r_* = 926.45	*D*_x_ = 1.598 Mg m^−^^3^
Orthorhombic, *P**n**a*2_1_	Mo *K*α radiation, λ = 0.71073 Å
Hall symbol: P 2c -2n	Cell parameters from 9504 reflections
*a* = 9.2879 (2) Å	θ = 2.6–30.1°
*b* = 39.2730 (11) Å	µ = 3.20 mm^−^^1^
*c* = 10.5592 (3) Å	*T* = 100 K
*V* = 3851.61 (17) Å^3^	Irregular, colourless
*Z* = 4	0.50 × 0.42 × 0.34 mm

### Data collection

**Table:**

Bruker APEXII CCD area-detector diffractometer	10997 independent reflections
Radiation source: fine-focus sealed tube	10267 reflections with *I* > 2σ(*I*)
Graphite monochromator	*R*_int_ = 0.029
phi and ω scans	θ_max_ = 29.8°, θ_min_ = 2.3°
Absorption correction: multi-scan (*SADABS*; Bruker, 2008)	*h* = −12→12
*T*_min_ = 0.298, *T*_max_ = 0.410	*k* = −54→54
59694 measured reflections	*l* = −14→14

### Refinement

**Table:**

Refinement on *F*^2^	Secondary atom site location: difference Fourier map
Least-squares matrix: full	Hydrogen site location: inferred from neighbouring sites
*R*[*F*^2^ > 2σ(*F*^2^)] = 0.026	H-atom parameters constrained
*wR*(*F*^2^) = 0.057	*w* = 1/[σ^2^(*F*_o_^2^) + (0.0167*P*)^2^ + 1.9141*P*] where *P* = (*F*_o_^2^ + 2*F*_c_^2^)/3
*S* = 1.06	(Δ/σ)_max_ = 0.001
10997 reflections	Δρ_max_ = 0.41 e Å^−^^3^
482 parameters	Δρ_min_ = −0.43 e Å^−^^3^
1 restraint	Absolute structure: Flack (1983), 5205 Friedel pairs
Primary atom site location: structure-invariant direct methods	Absolute structure parameter: 0.015 (4)

### Special details

**Table:**

Geometry. All e.s.d.'s (except the e.s.d. in the dihedral angle between two l.s. planes) are estimated using the full covariance matrix. The cell e.s.d.'s are taken into account individually in the estimation of e.s.d.'s in distances, angles and torsion angles; correlations between e.s.d.'s in cell parameters are only used when they are defined by crystal symmetry. An approximate (isotropic) treatment of cell e.s.d.'s is used for estimating e.s.d.'s involving l.s. planes.
Refinement. Refinement of *F*^2^ against ALL reflections. The weighted *R*-factor *wR* and goodness of fit *S* are based on *F*^2^, conventional *R*-factors *R* are based on *F*, with *F* set to zero for negative *F*^2^. The threshold expression of *F*^2^ > σ(*F*^2^) is used only for calculating *R*-factors(gt) *etc*. and is not relevant to the choice of reflections for refinement. *R*-factors based on *F*^2^ are statistically about twice as large as those based on *F*, and *R*- factors based on ALL data will be even larger.

### Fractional atomic coordinates and isotropic or equivalent isotropic displacement parameters (Å^2^)

**Table:**

	*x*	*y*	*z*	*U*_iso_*/*U*_eq_	
Br1	0.87579 (2)	0.101954 (5)	−0.01024 (2)	0.02056 (5)	
Br2	1.31949 (2)	0.203008 (5)	0.23399 (2)	0.01976 (4)	
Br3	0.62645 (2)	0.184625 (6)	0.55424 (2)	0.02518 (5)	
O1	0.62966 (17)	0.18819 (4)	0.09052 (18)	0.0237 (4)	
O2	0.50556 (16)	0.07721 (3)	0.15611 (16)	0.0186 (3)	
O3	1.16142 (18)	0.04624 (4)	0.10085 (18)	0.0278 (4)	
O4	1.3171 (2)	0.10168 (4)	0.45440 (17)	0.0289 (4)	
O5	1.10398 (16)	0.16994 (4)	0.62809 (16)	0.0188 (3)	
O6	0.81813 (16)	0.26402 (4)	0.58804 (16)	0.0197 (3)	
N1	0.58624 (17)	0.13273 (4)	0.15120 (17)	0.0138 (3)	
N2	1.20917 (17)	0.08138 (4)	0.27224 (17)	0.0144 (3)	
N3	0.97292 (17)	0.21791 (4)	0.57551 (16)	0.0123 (3)	
C1	0.79776 (19)	0.14557 (5)	0.29093 (19)	0.0118 (4)	
C2	0.90990 (19)	0.12503 (4)	0.2461 (2)	0.0120 (3)	
C3	1.0543 (2)	0.13449 (5)	0.26749 (18)	0.0117 (4)	
C4	1.0854 (2)	0.16428 (5)	0.33462 (19)	0.0113 (4)	
C5	0.9733 (2)	0.18575 (4)	0.37434 (19)	0.0109 (4)	
C6	0.8299 (2)	0.17643 (5)	0.35213 (19)	0.0120 (4)	
C7	0.8773 (2)	0.09313 (5)	0.1739 (2)	0.0146 (4)	
H7A	0.7823	0.0842	0.2003	0.017*	
H7B	0.9508	0.0757	0.1935	0.017*	
C8	1.2373 (2)	0.17347 (5)	0.3659 (2)	0.0132 (4)	
H8A	1.2957	0.1525	0.3733	0.016*	
H8B	1.2401	0.1854	0.4484	0.016*	
C9	0.7111 (2)	0.19981 (5)	0.3924 (2)	0.0164 (4)	
H9A	0.6356	0.2003	0.3263	0.020*	
H9B	0.7494	0.2232	0.4021	0.020*	
C10	0.6420 (2)	0.13404 (5)	0.2795 (2)	0.0144 (4)	
H10A	0.6332	0.1111	0.3176	0.017*	
H10B	0.5810	0.1497	0.3297	0.017*	
C11	0.5808 (2)	0.16045 (5)	0.0676 (2)	0.0181 (4)	
C12	0.5030 (2)	0.14809 (6)	−0.0454 (2)	0.0193 (4)	
C13	0.4667 (2)	0.16503 (7)	−0.1558 (2)	0.0302 (6)	
H13	0.4953	0.1879	−0.1703	0.036*	
C14	0.3862 (2)	0.14693 (9)	−0.2448 (3)	0.0396 (7)	
H14	0.3603	0.1577	−0.3221	0.048*	
C15	0.3432 (3)	0.11369 (9)	−0.2229 (3)	0.0413 (8)	
H15	0.2870	0.1022	−0.2848	0.050*	
C16	0.3809 (2)	0.09660 (8)	−0.1111 (3)	0.0302 (6)	
H16	0.3523	0.0738	−0.0957	0.036*	
C17	0.4624 (2)	0.11481 (6)	−0.0243 (2)	0.0196 (4)	
C18	0.5164 (2)	0.10438 (5)	0.1015 (2)	0.0151 (4)	
C19	1.1748 (2)	0.11348 (5)	0.20873 (19)	0.0132 (4)	
H19A	1.1484	0.1084	0.1199	0.016*	
H19B	1.2630	0.1276	0.2067	0.016*	
C20	1.2076 (2)	0.05012 (5)	0.2068 (2)	0.0194 (5)	
C21	1.2715 (2)	0.02478 (5)	0.2940 (2)	0.0220 (5)	
C22	1.2896 (2)	−0.01012 (6)	0.2806 (3)	0.0313 (6)	
H22	1.2603	−0.0217	0.2059	0.038*	
C23	1.3520 (3)	−0.02728 (6)	0.3804 (4)	0.0386 (7)	
H23	1.3643	−0.0512	0.3749	0.046*	
C24	1.3972 (3)	−0.01038 (6)	0.4888 (4)	0.0392 (7)	
H24	1.4407	−0.0229	0.5555	0.047*	
C25	1.3799 (3)	0.02466 (6)	0.5013 (3)	0.0327 (6)	
H25	1.4109	0.0364	0.5751	0.039*	
C26	1.3160 (2)	0.04157 (5)	0.4020 (2)	0.0223 (5)	
C27	1.2844 (2)	0.07837 (5)	0.3867 (2)	0.0191 (4)	
C28	1.0073 (2)	0.21895 (5)	0.44127 (19)	0.0124 (4)	
H28A	1.1110	0.2241	0.4309	0.015*	
H28B	0.9520	0.2376	0.4009	0.015*	
C29	1.0301 (2)	0.19385 (5)	0.6595 (2)	0.0148 (4)	
C30	0.9806 (2)	0.20383 (5)	0.7871 (2)	0.0170 (4)	
C31	1.0109 (2)	0.18917 (6)	0.9036 (2)	0.0217 (5)	
H31	1.0716	0.1698	0.9110	0.026*	
C32	0.9476 (3)	0.20435 (6)	1.0092 (2)	0.0292 (5)	
H32	0.9658	0.1953	1.0911	0.035*	
C33	0.8587 (3)	0.23229 (6)	0.9963 (3)	0.0299 (5)	
H33	0.8164	0.2419	1.0701	0.036*	
C34	0.8289 (3)	0.24692 (6)	0.8796 (2)	0.0232 (5)	
H34	0.7676	0.2662	0.8721	0.028*	
C35	0.8927 (2)	0.23217 (5)	0.7747 (2)	0.0172 (4)	
C36	0.8850 (2)	0.24137 (5)	0.6388 (2)	0.0155 (4)	
C1A	0.8364 (2)	0.03354 (5)	0.44059 (17)	0.0507 (9)	
C2A	0.7319 (2)	0.05829 (5)	0.4604 (2)	0.0498 (8)	
H2A	0.6435	0.0573	0.4151	0.060*	
C3A	0.7565 (3)	0.08446 (5)	0.5463 (2)	0.0810 (14)	
H3A	0.6850	0.1014	0.5598	0.097*	
C4A	0.8858 (4)	0.08588 (6)	0.6124 (2)	0.0887 (18)	
H4A	0.9026	0.1038	0.6712	0.106*	
C5A	0.9904 (3)	0.06113 (8)	0.5927 (2)	0.0813 (15)	
H5A	1.0787	0.0621	0.6379	0.098*	
C6A	0.96570 (19)	0.03496 (7)	0.5067 (2)	0.0693 (12)	
H6A	1.0372	0.0180	0.4932	0.083*	
C7A	0.8255 (4)	0.00470 (6)	0.3449 (2)	0.0941 (18)	
H7A1	0.7240	−0.0012	0.3319	0.141*	
H7A2	0.8776	−0.0152	0.3767	0.141*	
H7A3	0.8677	0.0120	0.2642	0.141*	

### Atomic displacement parameters (Å^2^)

**Table:**

	*U*^11^	*U*^22^	*U*^33^	*U*^12^	*U*^13^	*U*^23^
Br1	0.02090 (10)	0.02545 (10)	0.01532 (10)	−0.00046 (8)	−0.00065 (9)	−0.00518 (9)
Br2	0.01633 (8)	0.02241 (9)	0.02055 (10)	−0.00556 (8)	0.00482 (9)	0.00259 (9)
Br3	0.01890 (10)	0.03088 (11)	0.02576 (12)	−0.00392 (8)	0.01001 (10)	−0.01086 (10)
O1	0.0196 (7)	0.0195 (7)	0.0320 (10)	0.0003 (6)	0.0050 (7)	0.0052 (7)
O2	0.0196 (7)	0.0134 (6)	0.0228 (8)	−0.0023 (5)	0.0005 (6)	−0.0051 (6)
O3	0.0283 (9)	0.0211 (8)	0.0340 (10)	0.0060 (6)	−0.0097 (7)	−0.0118 (7)
O4	0.0437 (11)	0.0207 (8)	0.0223 (9)	0.0049 (7)	−0.0119 (7)	−0.0006 (7)
O5	0.0186 (7)	0.0170 (7)	0.0208 (8)	0.0065 (5)	0.0003 (6)	0.0032 (6)
O6	0.0195 (7)	0.0140 (7)	0.0255 (9)	0.0049 (5)	−0.0014 (6)	−0.0022 (6)
N1	0.0108 (8)	0.0153 (7)	0.0152 (9)	−0.0007 (6)	−0.0010 (6)	−0.0015 (6)
N2	0.0132 (8)	0.0125 (7)	0.0176 (9)	0.0024 (6)	0.0003 (6)	−0.0013 (6)
N3	0.0141 (7)	0.0108 (7)	0.0120 (9)	0.0015 (6)	−0.0002 (6)	−0.0023 (6)
C1	0.0089 (8)	0.0136 (8)	0.0130 (10)	−0.0002 (6)	−0.0009 (7)	0.0008 (7)
C2	0.0125 (8)	0.0120 (8)	0.0114 (9)	−0.0012 (6)	0.0001 (7)	0.0007 (7)
C3	0.0121 (8)	0.0107 (8)	0.0122 (9)	0.0024 (6)	−0.0002 (7)	0.0019 (7)
C4	0.0099 (8)	0.0124 (8)	0.0116 (10)	0.0004 (6)	−0.0004 (7)	0.0025 (7)
C5	0.0103 (8)	0.0105 (8)	0.0119 (10)	−0.0008 (6)	−0.0003 (7)	0.0011 (7)
C6	0.0105 (8)	0.0134 (8)	0.0121 (9)	0.0014 (6)	−0.0008 (7)	−0.0012 (7)
C7	0.0154 (9)	0.0129 (8)	0.0153 (10)	−0.0006 (7)	−0.0003 (8)	−0.0021 (7)
C8	0.0110 (8)	0.0133 (8)	0.0153 (10)	−0.0033 (7)	0.0005 (7)	0.0013 (7)
C9	0.0114 (9)	0.0165 (9)	0.0211 (11)	0.0014 (7)	−0.0001 (8)	−0.0059 (8)
C10	0.0100 (8)	0.0182 (9)	0.0148 (10)	−0.0022 (7)	−0.0003 (7)	−0.0027 (7)
C11	0.0116 (8)	0.0216 (9)	0.0211 (11)	0.0037 (7)	0.0046 (8)	0.0024 (9)
C12	0.0113 (9)	0.0303 (11)	0.0163 (11)	0.0051 (8)	0.0026 (7)	0.0028 (8)
C13	0.0156 (10)	0.0522 (15)	0.0228 (13)	0.0093 (10)	0.0039 (9)	0.0130 (11)
C14	0.0174 (11)	0.082 (2)	0.0198 (13)	0.0076 (12)	−0.0012 (10)	0.0112 (13)
C15	0.0153 (11)	0.089 (2)	0.0190 (13)	−0.0030 (12)	−0.0040 (9)	−0.0087 (14)
C16	0.0153 (11)	0.0512 (15)	0.0242 (13)	−0.0040 (10)	−0.0011 (9)	−0.0113 (11)
C17	0.0124 (9)	0.0274 (10)	0.0189 (11)	0.0051 (7)	−0.0001 (8)	−0.0066 (9)
C18	0.0091 (8)	0.0188 (9)	0.0176 (10)	0.0018 (7)	0.0017 (7)	−0.0067 (8)
C19	0.0122 (8)	0.0132 (8)	0.0142 (11)	0.0009 (7)	0.0015 (7)	−0.0003 (7)
C20	0.0141 (9)	0.0149 (9)	0.0291 (14)	0.0011 (7)	0.0005 (8)	−0.0034 (8)
C21	0.0149 (9)	0.0156 (9)	0.0354 (14)	0.0010 (7)	0.0021 (9)	0.0019 (9)
C22	0.0228 (11)	0.0169 (10)	0.0542 (18)	0.0028 (8)	−0.0008 (11)	0.0001 (10)
C23	0.0284 (13)	0.0144 (10)	0.073 (2)	0.0023 (9)	−0.0004 (13)	0.0116 (12)
C24	0.0355 (14)	0.0264 (12)	0.0558 (19)	0.0055 (10)	−0.0055 (14)	0.0185 (14)
C25	0.0336 (13)	0.0279 (11)	0.0366 (15)	0.0050 (10)	0.0006 (12)	0.0129 (11)
C26	0.0188 (10)	0.0174 (9)	0.0306 (13)	0.0021 (8)	0.0019 (9)	0.0067 (9)
C27	0.0195 (10)	0.0190 (9)	0.0189 (11)	0.0036 (8)	−0.0002 (8)	0.0018 (8)
C28	0.0123 (9)	0.0120 (8)	0.0128 (9)	−0.0003 (7)	−0.0016 (7)	0.0002 (7)
C29	0.0119 (9)	0.0169 (9)	0.0157 (10)	−0.0012 (7)	−0.0012 (8)	0.0018 (8)
C30	0.0155 (9)	0.0205 (10)	0.0150 (10)	−0.0058 (8)	−0.0004 (8)	−0.0003 (8)
C31	0.0207 (11)	0.0261 (11)	0.0184 (11)	−0.0059 (8)	−0.0015 (9)	0.0039 (9)
C32	0.0363 (14)	0.0348 (13)	0.0164 (12)	−0.0132 (10)	0.0043 (10)	−0.0003 (9)
C33	0.0392 (14)	0.0307 (11)	0.0197 (12)	−0.0137 (10)	0.0120 (11)	−0.0092 (11)
C34	0.0257 (11)	0.0197 (10)	0.0241 (13)	−0.0056 (8)	0.0079 (10)	−0.0081 (9)
C35	0.0161 (9)	0.0172 (9)	0.0182 (11)	−0.0049 (7)	0.0005 (8)	−0.0051 (8)
C36	0.0127 (9)	0.0134 (8)	0.0202 (11)	−0.0013 (7)	−0.0001 (8)	−0.0048 (8)
C1A	0.055 (2)	0.059 (2)	0.0382 (19)	−0.0185 (16)	0.0039 (15)	0.0224 (16)
C2A	0.057 (2)	0.0499 (18)	0.042 (2)	−0.0087 (15)	−0.0076 (15)	0.0249 (15)
C3A	0.150 (5)	0.045 (2)	0.048 (2)	0.016 (2)	0.005 (3)	0.0289 (19)
C4A	0.180 (6)	0.055 (2)	0.032 (2)	−0.052 (3)	−0.004 (3)	0.0074 (18)
C5A	0.077 (3)	0.096 (3)	0.071 (3)	−0.053 (3)	−0.007 (2)	0.020 (3)
C6A	0.0417 (19)	0.109 (3)	0.057 (3)	−0.019 (2)	0.0108 (17)	0.028 (2)
C7A	0.171 (6)	0.058 (2)	0.053 (3)	−0.017 (3)	0.016 (3)	0.011 (2)

### Geometric parameters (Å, º)

**Table:**

Br1—C7	1.975 (2)	C16—C17	1.387 (3)
Br2—C8	1.967 (2)	C16—H16	0.9500
Br3—C9	1.974 (2)	C17—C18	1.478 (3)
O1—C11	1.205 (3)	C19—H19A	0.9900
O2—C18	1.217 (3)	C19—H19B	0.9900
O3—C20	1.208 (3)	C20—C21	1.480 (3)
O4—C27	1.201 (3)	C21—C26	1.380 (3)
O5—C29	1.209 (2)	C21—C22	1.388 (3)
O6—C36	1.210 (2)	C22—C23	1.379 (4)
N1—C18	1.391 (2)	C22—H22	0.9500
N1—C11	1.403 (3)	C23—C24	1.388 (5)
N1—C10	1.451 (3)	C23—H23	0.9500
N2—C27	1.401 (3)	C24—C25	1.392 (4)
N2—C20	1.409 (3)	C24—H24	0.9500
N2—C19	1.463 (2)	C25—C26	1.376 (4)
N3—C36	1.401 (2)	C25—H25	0.9500
N3—C29	1.401 (3)	C26—C27	1.484 (3)
N3—C28	1.454 (3)	C28—H28A	0.9900
C1—C2	1.400 (3)	C28—H28B	0.9900
C1—C6	1.405 (3)	C29—C30	1.477 (3)
C1—C10	1.521 (3)	C30—C35	1.387 (3)
C2—C3	1.410 (2)	C30—C31	1.387 (3)
C2—C7	1.498 (3)	C31—C32	1.395 (4)
C3—C4	1.398 (3)	C31—H31	0.9500
C3—C19	1.523 (3)	C32—C33	1.380 (4)
C4—C5	1.404 (3)	C32—H32	0.9500
C4—C8	1.494 (3)	C33—C34	1.388 (4)
C5—C6	1.401 (3)	C33—H33	0.9500
C5—C28	1.517 (3)	C34—C35	1.383 (3)
C6—C9	1.496 (3)	C34—H34	0.9500
C7—H7A	0.9900	C35—C36	1.482 (3)
C7—H7B	0.9900	C1A—C2A	1.3900
C8—H8A	0.9900	C1A—C6A	1.3900
C8—H8B	0.9900	C1A—C7A	1.5213
C9—H9A	0.9900	C2A—C3A	1.3900
C9—H9B	0.9900	C2A—H2A	0.9500
C10—H10A	0.9900	C3A—C4A	1.3900
C10—H10B	0.9900	C3A—H3A	0.9500
C11—C12	1.476 (3)	C4A—C5A	1.3900
C12—C17	1.378 (3)	C4A—H4A	0.9500
C12—C13	1.384 (3)	C5A—C6A	1.3900
C13—C14	1.396 (4)	C5A—H5A	0.9500
C13—H13	0.9500	C6A—H6A	0.9500
C14—C15	1.385 (5)	C7A—H7A1	0.9800
C14—H14	0.9500	C7A—H7A2	0.9800
C15—C16	1.402 (4)	C7A—H7A3	0.9800
C15—H15	0.9500		
			
C18—N1—C11	111.52 (18)	H19A—C19—H19B	107.4
C18—N1—C10	123.14 (17)	O3—C20—N2	124.61 (19)
C11—N1—C10	124.97 (17)	O3—C20—C21	129.3 (2)
C27—N2—C20	110.78 (17)	N2—C20—C21	106.06 (19)
C27—N2—C19	125.23 (16)	C26—C21—C22	121.3 (2)
C20—N2—C19	121.58 (17)	C26—C21—C20	108.21 (18)
C36—N3—C29	111.29 (17)	C22—C21—C20	130.5 (2)
C36—N3—C28	125.06 (17)	C23—C22—C21	117.1 (3)
C29—N3—C28	123.58 (16)	C23—C22—H22	121.5
C2—C1—C6	119.64 (16)	C21—C22—H22	121.5
C2—C1—C10	120.62 (17)	C22—C23—C24	121.6 (2)
C6—C1—C10	119.67 (17)	C22—C23—H23	119.2
C1—C2—C3	120.11 (17)	C24—C23—H23	119.2
C1—C2—C7	120.26 (16)	C23—C24—C25	121.1 (3)
C3—C2—C7	119.63 (16)	C23—C24—H24	119.5
C4—C3—C2	119.88 (17)	C25—C24—H24	119.5
C4—C3—C19	120.55 (16)	C26—C25—C24	117.1 (3)
C2—C3—C19	119.42 (17)	C26—C25—H25	121.5
C3—C4—C5	120.07 (17)	C24—C25—H25	121.5
C3—C4—C8	120.62 (17)	C25—C26—C21	121.9 (2)
C5—C4—C8	119.31 (17)	C25—C26—C27	129.7 (2)
C6—C5—C4	119.88 (17)	C21—C26—C27	108.4 (2)
C6—C5—C28	120.05 (16)	O4—C27—N2	125.15 (19)
C4—C5—C28	120.07 (16)	O4—C27—C26	128.9 (2)
C5—C6—C1	120.26 (17)	N2—C27—C26	105.95 (19)
C5—C6—C9	119.51 (17)	N3—C28—C5	112.61 (16)
C1—C6—C9	120.22 (17)	N3—C28—H28A	109.1
C2—C7—Br1	110.86 (14)	C5—C28—H28A	109.1
C2—C7—H7A	109.5	N3—C28—H28B	109.1
Br1—C7—H7A	109.5	C5—C28—H28B	109.1
C2—C7—H7B	109.5	H28A—C28—H28B	107.8
Br1—C7—H7B	109.5	O5—C29—N3	124.4 (2)
H7A—C7—H7B	108.1	O5—C29—C30	129.29 (19)
C4—C8—Br2	110.66 (14)	N3—C29—C30	106.26 (16)
C4—C8—H8A	109.5	C35—C30—C31	122.4 (2)
Br2—C8—H8A	109.5	C35—C30—C29	108.11 (18)
C4—C8—H8B	109.5	C31—C30—C29	129.5 (2)
Br2—C8—H8B	109.5	C30—C31—C32	116.5 (2)
H8A—C8—H8B	108.1	C30—C31—H31	121.8
C6—C9—Br3	110.74 (14)	C32—C31—H31	121.8
C6—C9—H9A	109.5	C33—C32—C31	120.9 (2)
Br3—C9—H9A	109.5	C33—C32—H32	119.5
C6—C9—H9B	109.5	C31—C32—H32	119.5
Br3—C9—H9B	109.5	C32—C33—C34	122.4 (2)
H9A—C9—H9B	108.1	C32—C33—H33	118.8
N1—C10—C1	115.09 (17)	C34—C33—H33	118.8
N1—C10—H10A	108.5	C35—C34—C33	116.9 (2)
C1—C10—H10A	108.5	C35—C34—H34	121.6
N1—C10—H10B	108.5	C33—C34—H34	121.6
C1—C10—H10B	108.5	C34—C35—C30	120.9 (2)
H10A—C10—H10B	107.5	C34—C35—C36	130.8 (2)
O1—C11—N1	124.2 (2)	C30—C35—C36	108.36 (18)
O1—C11—C12	130.1 (2)	O6—C36—N3	124.9 (2)
N1—C11—C12	105.73 (17)	O6—C36—C35	129.2 (2)
C17—C12—C13	121.7 (2)	N3—C36—C35	105.89 (17)
C17—C12—C11	108.37 (19)	C2A—C1A—C6A	120.0
C13—C12—C11	129.9 (2)	C2A—C1A—C7A	125.0
C12—C13—C14	116.9 (3)	C6A—C1A—C7A	114.9
C12—C13—H13	121.5	C3A—C2A—C1A	120.0
C14—C13—H13	121.5	C3A—C2A—H2A	120.0
C15—C14—C13	121.5 (3)	C1A—C2A—H2A	120.0
C15—C14—H14	119.3	C2A—C3A—C4A	120.0
C13—C14—H14	119.3	C2A—C3A—H3A	120.0
C14—C15—C16	121.3 (3)	C4A—C3A—H3A	120.0
C14—C15—H15	119.4	C5A—C4A—C3A	120.0
C16—C15—H15	119.3	C5A—C4A—H4A	120.0
C17—C16—C15	116.5 (3)	C3A—C4A—H4A	120.0
C17—C16—H16	121.8	C6A—C5A—C4A	120.0
C15—C16—H16	121.8	C6A—C5A—H5A	120.0
C12—C17—C16	122.1 (2)	C4A—C5A—H5A	120.0
C12—C17—C18	108.37 (19)	C5A—C6A—C1A	120.0
C16—C17—C18	129.5 (2)	C5A—C6A—H6A	120.0
O2—C18—N1	124.2 (2)	C1A—C6A—H6A	120.0
O2—C18—C17	129.84 (19)	C1A—C7A—H7A1	109.5
N1—C18—C17	105.98 (17)	C1A—C7A—H7A2	109.5
N2—C19—C3	116.12 (16)	H7A1—C7A—H7A2	109.5
N2—C19—H19A	108.3	C1A—C7A—H7A3	109.5
C3—C19—H19A	108.3	H7A1—C7A—H7A3	109.5
N2—C19—H19B	108.3	H7A2—C7A—H7A3	109.5
C3—C19—H19B	108.3		
			
C6—C1—C2—C3	3.0 (3)	C19—N2—C20—O3	−9.9 (3)
C10—C1—C2—C3	−174.19 (18)	C27—N2—C20—C21	7.1 (2)
C6—C1—C2—C7	−176.32 (19)	C19—N2—C20—C21	170.34 (17)
C10—C1—C2—C7	6.5 (3)	O3—C20—C21—C26	176.7 (2)
C1—C2—C3—C4	0.6 (3)	N2—C20—C21—C26	−3.6 (2)
C7—C2—C3—C4	179.86 (18)	O3—C20—C21—C22	−3.3 (4)
C1—C2—C3—C19	−174.93 (18)	N2—C20—C21—C22	176.4 (2)
C7—C2—C3—C19	4.3 (3)	C26—C21—C22—C23	0.9 (4)
C2—C3—C4—C5	−3.7 (3)	C20—C21—C22—C23	−179.1 (2)
C19—C3—C4—C5	171.81 (18)	C21—C22—C23—C24	−1.1 (4)
C2—C3—C4—C8	175.78 (18)	C22—C23—C24—C25	0.6 (4)
C19—C3—C4—C8	−8.8 (3)	C23—C24—C25—C26	0.2 (4)
C3—C4—C5—C6	3.2 (3)	C24—C25—C26—C21	−0.4 (4)
C8—C4—C5—C6	−176.28 (18)	C24—C25—C26—C27	−179.3 (2)
C3—C4—C5—C28	−176.88 (18)	C22—C21—C26—C25	−0.1 (4)
C8—C4—C5—C28	3.7 (3)	C20—C21—C26—C25	179.9 (2)
C4—C5—C6—C1	0.4 (3)	C22—C21—C26—C27	179.0 (2)
C28—C5—C6—C1	−179.56 (18)	C20—C21—C26—C27	−1.0 (2)
C4—C5—C6—C9	−178.73 (19)	C20—N2—C27—O4	170.9 (2)
C28—C5—C6—C9	1.3 (3)	C19—N2—C27—O4	8.4 (3)
C2—C1—C6—C5	−3.4 (3)	C20—N2—C27—C26	−7.7 (2)
C10—C1—C6—C5	173.72 (18)	C19—N2—C27—C26	−170.18 (18)
C2—C1—C6—C9	175.67 (19)	C25—C26—C27—O4	5.7 (4)
C10—C1—C6—C9	−7.2 (3)	C21—C26—C27—O4	−173.3 (2)
C1—C2—C7—Br1	93.3 (2)	C25—C26—C27—N2	−175.8 (2)
C3—C2—C7—Br1	−85.9 (2)	C21—C26—C27—N2	5.3 (2)
C3—C4—C8—Br2	92.48 (19)	C36—N3—C28—C5	−126.16 (18)
C5—C4—C8—Br2	−88.1 (2)	C29—N3—C28—C5	57.0 (2)
C5—C6—C9—Br3	−99.78 (19)	C6—C5—C28—N3	72.1 (2)
C1—C6—C9—Br3	81.1 (2)	C4—C5—C28—N3	−107.8 (2)
C18—N1—C10—C1	129.25 (19)	C36—N3—C29—O5	176.44 (19)
C11—N1—C10—C1	−58.3 (2)	C28—N3—C29—O5	−6.3 (3)
C2—C1—C10—N1	−68.4 (2)	C36—N3—C29—C30	−3.0 (2)
C6—C1—C10—N1	114.4 (2)	C28—N3—C29—C30	174.23 (16)
C18—N1—C11—O1	177.67 (19)	O5—C29—C30—C35	−177.2 (2)
C10—N1—C11—O1	4.5 (3)	N3—C29—C30—C35	2.2 (2)
C18—N1—C11—C12	−1.2 (2)	O5—C29—C30—C31	3.0 (4)
C10—N1—C11—C12	−174.34 (17)	N3—C29—C30—C31	−177.6 (2)
O1—C11—C12—C17	−177.0 (2)	C35—C30—C31—C32	0.3 (3)
N1—C11—C12—C17	1.7 (2)	C29—C30—C31—C32	−179.9 (2)
O1—C11—C12—C13	0.7 (4)	C30—C31—C32—C33	0.5 (3)
N1—C11—C12—C13	179.4 (2)	C31—C32—C33—C34	−0.7 (4)
C17—C12—C13—C14	0.3 (3)	C32—C33—C34—C35	0.0 (3)
C11—C12—C13—C14	−177.1 (2)	C33—C34—C35—C30	0.8 (3)
C12—C13—C14—C15	0.7 (4)	C33—C34—C35—C36	−179.4 (2)
C13—C14—C15—C16	−1.1 (4)	C31—C30—C35—C34	−1.0 (3)
C14—C15—C16—C17	0.4 (4)	C29—C30—C35—C34	179.18 (19)
C13—C12—C17—C16	−1.1 (3)	C31—C30—C35—C36	179.16 (19)
C11—C12—C17—C16	176.8 (2)	C29—C30—C35—C36	−0.7 (2)
C13—C12—C17—C18	−179.57 (19)	C29—N3—C36—O6	−177.11 (19)
C11—C12—C17—C18	−1.6 (2)	C28—N3—C36—O6	5.7 (3)
C15—C16—C17—C12	0.7 (3)	C29—N3—C36—C35	2.6 (2)
C15—C16—C17—C18	178.8 (2)	C28—N3—C36—C35	−174.60 (17)
C11—N1—C18—O2	−179.56 (19)	C34—C35—C36—O6	−1.3 (4)
C10—N1—C18—O2	−6.2 (3)	C30—C35—C36—O6	178.6 (2)
C11—N1—C18—C17	0.2 (2)	C34—C35—C36—N3	179.1 (2)
C10—N1—C18—C17	173.51 (17)	C30—C35—C36—N3	−1.1 (2)
C12—C17—C18—O2	−179.3 (2)	C6A—C1A—C2A—C3A	0.0
C16—C17—C18—O2	2.4 (4)	C7A—C1A—C2A—C3A	−176.9
C12—C17—C18—N1	0.9 (2)	C1A—C2A—C3A—C4A	0.0
C16—C17—C18—N1	−177.4 (2)	C2A—C3A—C4A—C5A	0.0
C27—N2—C19—C3	−74.7 (2)	C3A—C4A—C5A—C6A	0.0
C20—N2—C19—C3	124.55 (19)	C4A—C5A—C6A—C1A	0.0
C4—C3—C19—N2	106.5 (2)	C2A—C1A—C6A—C5A	0.0
C2—C3—C19—N2	−78.0 (2)	C7A—C1A—C6A—C5A	177.2
C27—N2—C20—O3	−173.1 (2)		

### Hydrogen-bond geometry (Å, º)

Cg1 and Cg2 are centroids of the C1A–C6A and C21–C26 benzene rings, respectively.

**Table:**

*D*—H···*A*	*D*—H	H···*A*	*D*···*A*	*D*—H···*A*
C7—H7*B*···O3	0.99	2.47	3.309 (3)	142
C8—H8*A*···O4	0.99	2.18	3.061 (3)	147
C8—H8*B*···O5	0.99	2.36	3.036 (3)	125
C8—H8*B*···O6^i^	0.99	2.58	3.477 (3)	151
C9—H9*A*···O1	0.99	2.54	3.308 (3)	135
C10—H10*B*···Br3	0.99	2.77	3.519 (2)	133
C14—H14···O5^ii^	0.95	2.49	3.080 (3)	121
C19—H19*A*···Br1	0.99	2.89	3.642 (2)	133
C24—H24···O2^iii^	0.95	2.43	3.290 (3)	150
C28—H28*A*···O6^i^	0.99	2.58	3.344 (2)	134
C33—H33···Br2^iv^	0.95	2.77	3.590 (3)	145
C22—H22···*Cg*1^v^	0.95	2.66	3.599 (3)	169
C6*A*—H6*A*···*Cg*2	0.95	2.99	3.795 (3)	143

Symmetry codes: (i) *x*+1/2, −*y*+1/2, *z*; (ii) *x*−1, *y*, *z*−1; (iii) −*x*+2, −*y*, *z*+1/2; (iv) *x*−1/2, −*y*+1/2, *z*+1; (v) −*x*+2, −*y*−1, *z*+1/2.
